# Panel Practice and the Future of Partnerships

**Published:** 1913-09-06

**Authors:** 


### PANEL PRACTICE AND THE FUTURE OF PARTNERSHIPS.

It is unfortunate the authors of the Insurance Act ignored the fact that the conditions of medical practice are not the same throughout Britain. It is in poor agricultural districts that the panel system works with least friction because the doctor finds himself, on the whole, better off and the patients feel little change in the conditions. The doctor is better off because he gets more money per head for the men who were on the club lists before the Act was passed, and he also gets payment for many men who were not formerly in any club, and who either paid nothing when they did call in the doctor or called him in on an order obtained from the relieving officer. Since the salary of the parish doctors has not been reduced by the passing of the Act the practitioner who was parish and club doctor in an agricultural community gains by the panel system to the extent indicated. And in such districts his gains on that count are but little reduced by the losing of the small bills of people with about £100 a year, who used to pay for medical attendance and who now are contract patients. The abolition of the books and the substitution of cards have removed much clerical work which was and is disliked by panel doctors of every sort. So in such districts the doctors gain usually, and since the patients are still attended in the same way by the men to whom they have been accustomed they have little cause for complaint.

More of the doubtful blessing of guinea midwifery seems to be coming to the panel doctors in rural districts. To get the maternity benefit the certificate of a medical man or of a qualified midwife is necessary. So one or the other must be called in. Qualified midwives are not plentiful in rural districts, and those there are charge little, if any, less than the doctor. The women in country districts still have a prejudice in favour of a man to attend them in labour. The upshot is that women who formerly relied on the ministrations of the wise woman of the village now seem inclined to call in the panel doctor. In spite of the advantages gained by the doctors in these districts, there is an uneasy feeling throughout them that the takings will not prove to be so good as they now promise to be. This uneasiness appears to be due to a distrust of the authorities.

In the large manufacturing towns and in mining districts things go less well. The position of the club doctor was always humiliating. The position of the panel doctor is worse. The men of these districts are often ignorant, usually bad mannered, and mostly members of a trade union. Because of their bad manners they liked to assert their superiority over their club doctor by treating him with scant respect or downright rudeness. When the doctors made their futile fight against the terms of the Insurance Act and stood out, or rather asserted that they meant to stand out, for better terms, they were condemned by the very men who stick at nothing in the promotion of the pecuniary interests of organised labour. There was a real fear that the doctors would insist on what they regarded as fair terms. The miners, mechanics, and artisans believed (or affected to believe) that the doctor's trade union had ordered a strike. They were angry when they thought that the men to whom they had thrown a pittance for medical attendance were likely to insist on better pay and more honourable conditions.

But when they saw doctors tumbling over one another to "blackleg" they despised the men who accepted the scheme. They did not understand that the club doctors had no organisation; had no penalties; had no trade union; had no means of bringing pressure to bear; had nothing to hold them together but promises.

This, with exaggeration granted, seems the position in the mining and manufacturing districts. The doctors feel a sense of injustice, angry indignation at the action the Government took to compel them to submit, and inward shame at the poor part the profession played in the eyes of the nation. Hence a want of conscientious working of the Act; a hope that it may break down; and this tends to perfunctory treatment of panel patients, to easy certificates, and a semi-conscious alliance with malingerers. The profession is demoralised, for the doctors, although often better off in the pecuniary sense, especially the men of low-class practice, see with indignation households that draw, or can draw if the members like to work full time, incomes as great as come to their own pockets, exploiting their skill for a few shillings per annum. The patients, finding that many of the most esteemed medical men stand out of the panel, conclude that the panel doctor is always an inferior man, and therefore feel that they are not being treated fairly. The best men seldom or never have taken a practice that was chiefly contract.

In residential districts, in good-class country practices, in good suburban districts panel practice makes little difference. The doctor goes on the panel as a convenience to his paying patients. His panel patients are chiefly the domestic servants and employees of his *clientèle*. He troubles little about cards, statistics, and red tape, because he does not care a cent whether he is on the panel or no, and he is perfectly well aware that the authorities will not dare to put off the panel a good man they have once roped in. Probably the authorities are already as well aware as the doctors that the statistics collected through the cards are not trustworthy, and their collection may be dropped.

The effect of panel practice on the capital value of practices is another matter that is subject for speculation. On the whole the takings of practices affected by the system seem to be greater, and certainly the riff-raff of the profession are greatly benefiting. But it seems likely that the selling price of practices and partnerships will be diminished. It is probably harder now to get a good price for a practice or partnership than it was a few years back. This is due to a diminished demand for practices and an increased demand for partners. The longer curriculum and the severity of examinations for qualification have lessened the supply of medical students, and consequently are lessening the percentage of doctors to population. There is an increase in the demand for medical men for all the Public Services. There is a larger emigration than formerly of qualified men to the Colonies. So it is more difficult to get good assistants or locum tenentes and very expensive to get any at all. Thus partners become necessities, and it is found to be good policy to offer a good man easy terms. This brings down the cash value of partnerships. It is obviously useless to expect a high price for a practice if you want to sell and there are few men thinking of buying. Dislike of being a panel doctor is said to be deterring many young men from joining the profession. If it be so it will be evident when the schools disclose their entries next October; and fewer students will produce still less demand for established practices.

The panel system in a more direct manner may cause a depreciation of cash values. Already there is an outcry that many men have too many panel patients. It is not possible for the Commissioners to refuse any man of decent repute a place on the panel. Once on the panel it will be easy to raise an agitation to have patients allotted to a newcomer if the established men have very large lists. So "squatting" may be more rapidly successful and more common since a considerable introduction may be rapidly obtained through panel patients. Men may prefer the chances of "squatting" in certain districts to the payment of premiums. This may make established practices less certain, further lessen the buyers, and knock down values. Partnerships may be still further depreciated if established men find that they must at all costs get a good partner to forestall a competitor.

The present unrest may result in a drawing together of the profession. On the whole this alternative seems most likely to happen. It is to be hoped it will happen. If it does not the medical profession is likely to split into two main castes—panellers and non-panellers—which cannot endure.

